# Mixing performance test and uniformity analysis of a two-stage injection jet on-line mixer

**DOI:** 10.1371/journal.pone.0326560

**Published:** 2025-06-23

**Authors:** Ping Jiang, Siliang Xiang, Wenwu Hu, Yahui Luo, Wen Li, Xiang Dong, Yixin Shi

**Affiliations:** 1 College of Mechanical and Electrical Engineering, Hunan Agricultural University, Changsha, China; 2 School of Agricultural Engineering, Jiangsu University, Zhenjiang, China; 3 Sunward Intelligent Equipment Co., Ltd., Changsha, China; NED University of Engineering and Technology, PAKISTAN

## Abstract

With the mixing of water and pesticides being a critical step, which is often performed using mixers. Traditional mixers, however, frequently exhibit issues such as uneven mixing and limited ratios. A dual-stage injection jet online mixer was designed to enhance the uniformity of pesticide and water mixing. This paper presents performance tests and uniformity analysis of this mixer, aiming to assess its effectiveness post-installation and to determine if it meets the required mixing performance and uniformity standards. Image analysis was utilized for qualitative assessment, revealing that the mixer’s two-dimensional curves in both axial and radial directions matched the pattern of pure pesticides. The root mean square error (RMSE) decreased with increasing mixing ratios, ranging from a minimum of 8.57% at a 300:1 ratio to a maximum of 9.94% at 2000:1. These variations were minimal, indicating good homogeneity across different ratios. Quantitative analysis using UV spectrophotometry showed that the actual mass concentration of the mixture deviated by less than 0.1% from the expected values at different sampling times, confirming superior temporal distribution uniformity. The variation in mixture mass concentration was insignificant across different locations, and the error variation at the same mixing ratio was less than 0.03%, indicating an excellent spatial distribution uniformity of the mixer. These findings demonstrate that the two-stage injection jet online mixer is effective over a wide range of mixing ratios, providing theoretical and technical support for accurate online variable spraying. This advancement is crucial for enhancing pesticide mixing efficiency and promoting sustainable agricultural development.

## Introduction

Precision variable plant protection operations are crucial for the development of modern agriculture. As a key piece of equipment, the mixer plays an indispensable role in precisely controlling the amount of pesticide sprayed and the mixing ratio. By achieving the precise operation mode of “mixing as much as spraying”, it can effectively reduce the waste and use of pesticides from the practical operation level. This not only helps to reduce agricultural production costs, but also alleviates the negative impact on the environment [1,2]. The existing pesticide water mixing methods are usually premixing and online mixing [3–7], and online mixing methods can be divided into two types: injection and jet-based methods [8–10]. The injection type uses external energy from the sprayer’s pipeline system to precisely extract pesticides and achieve mixing within the pipeline; while the jet type utilizes the energy transfer method of momentum exchange to accomplish the suction of pesticides and their mixing within the pipeline [11]. Jet mixers have the advantages of a simple structure, low cost and good mixing effect and are widely used in plant protection machinery; thus, jet mixers have attracted extensive attention from scientists and have been extensively developed and improved upon through continuous research.

Recent advances in geometric optimization of jet mixers have been achieved through computational fluid dynamics (CFD) simulations and machine learning algorithms, enabling enhanced design precision and performance prediction. Duan et al. [12] presents an optimized design of a multi-orifice-impinging transverse (MOIT) jet mixer by integrating the structures of a conventional MOIT and a venturi mixer, utilizing numerical simulations and experiments to validate its enhanced mixing efficiency and optimal operating parameters. Ou et al. [13] and other computational fluid dynamics (CFD) numerical simulation methods for jet mixing devices under variable operating conditions and flow characteristics were used in this study. The results showed that the numerical model can accurately predict the mixing ratio with changes in the static pressure at the outlet and that the mixing ratio at the outlet increases with linear reductions in the mixing ratio. Mohammadreza et al. [14] developed a data-driven optimization framework integrating computational fluid dynamics and machine learning, which achieved multidisciplinary optimization of geometric and operational parameters for rotor-stator mixers through limited high-fidelity CFD simulations, demonstrating a 20% improvement in mixing index and 50% reduction in shaft power consumption for high-viscosity fluids with marked superiority over conventional empirical design approaches. Xia et al. [15] conducted a numerical investigation of single-stage jet mixers using COMSOL Multiphysics, systematically analyzing the effects of area ratio, nozzle spacing, and inlet angle on mixing efficiency. Their work identified optimal parameter configurations that achieved a maximum coefficient of concentration variation of 8.20% over a mixing ratio range of 140:1–400:1, demonstrating favorable homogeneity. Ivana et al. [16] demonstrated a Bayesian optimization framework integrated with Gaussian processes to efficiently optimize geometric parameters of microfluidic mixers (e.g., parallelogrammic obstacles and Tesla-type configurations) within merely tens of numerical simulations, achieving at least an order-of-magnitude acceleration over conventional algorithms while significantly enhancing mixing performance. However, prior studies have demonstrated that conventional jet mixers exhibit limited mixing efficacy at low turndown ratios, with single-stage configurations typically constrained to a maximum operable range below 1000:1 due to inherent flow instabilities and incomplete turbulence dissipation.

Existing methodologies for evaluating mixing efficiency in mixers are broadly categorized into numerical simulations and experimental approaches. Egedy et al. [17] investigated the application of different jet geometries in pipelines to optimise the configuration of the jet mixer for effective homogenisation of the chemical composition and validated the quantitative and qualitative consistency of the model through experiments and simulations. Krupa [18] investigated the effect of the Reynolds coefficient on the performance of a mixer based on a microscopic method and found that increasing the ratio between the inlet diameter and the throat diameter improved the homogeneity of the studied mixture. Manjula et al. [19] investigated the effect of the operating parameters of a dual-jet mixer on the mixing time and predicted the mixing time with different parameters using magnitude analysis to establish the correlation between the properties of the dual-jet mixer and the mixing time. While existing studies predominantly focus on isolated parameter analysis, their accuracy and practicality in holistic evaluation remain constrained by the lack of integrative frameworks.

Recent advances in image-based analysis have enabled non-invasive, high-resolution characterization of mixing homogeneity, offering a transformative approach to quantify spatiotemporal uniformity in complex reactor systems. Liu et al. [20] established a quantitative pixel intensity-concentration correlation via light attenuation principles and developed a non-invasive method for density field mapping in confined flow systems. Chen [7] and others used high-speed camera and image processing technology combined with the principal component analysis method to study the on-line mixing flow fields under different flow rates and provided a scientific basis for the optimisation of the structure of mixers to maximise uniformity. However, this method is influenced by various factors, and it is not applicable under simple experimental conditions. Wang et al. [21] quantified liquid-liquid mixing homogeneity in microscale chaotic channels via image-based concentration field analysis, with CFD validation confirming strong experimental-simulated consistency. Metzger et al. [22] used a CFD method to simulate the transient flow characteristics of a jet mixing device and used laser-induced fluorescence and particle image velocimetry in experiments to validate the validity of the results of the CFD simulations. While image-based assessment techniques exhibit improved accuracy, their reliance on qualitative analysis, absence of standardized quantitative metrics, and neglect of system-level performance evaluation limit their robustness in comprehensive mixer characterization.

Conventional jet mixers suffer from inherent structural limitations that induce significant pressure energy losses, thereby degrading spray uniformity, while their constrained mixing ratio range (<1000:1) fails to ensure homogeneity under high-dilution conditions, particularly with pronounced flow disparity-induced concentration gradients. High-precision pesticides dilution (e.g., > 1000:1) is critical for optimizing pesticide dosage in large-scale field applications, yet existing mixers face persistent challenges in microcomponent metering accuracy and homogeneity maintenance under extreme mixing ratios, necessitating innovative designs with active flow control and extended ratio adaptability. In previous studies, a two-stage jet mixer with injective configuration was developed by employing CFD simulation and orthogonal design experiments. This mixer was configured to achieve a large mixing ratio through a two-stage cascaded jet mechanism, while an integrated injection system for pesticide and water was used to offset energy losses within the mixer. This design ultimately demonstrated the capability of achieving high flow rates, large mixing ratios, and precise mixing performance [23].

To scientifically evaluate the performance of the two-stage injection jet online mixer and thoroughly analyze its mixing uniformity, this study delved into the design principles and operational characteristics of the mixer. The mixing performance of the two-stage injection jet online mixer in plant protection operations was examined using high-speed photography image analysis [24]. The mixing time and spatial distribution uniformity of water-soluble pesticides mixed with water in the mixer were analysed with an approach based on ultraviolet spectrophotometry [25–28]. The aim of this study is to expand the mixing ratio of the mixer to 300:1–3000:1 with good uniformity, ensuring that the root mean square error (RMSE) of concentration at different mixing ratios is below 10%, and maintaining the relative error between the actual mass concentration and the expected value during operation below 0.1%. Through this research, we aim to provide theoretical references and technical support for achieving precise on-line variable spraying, offering an innovative spraying technique for pest control. This will ensure the effective application of pesticides on crops while significantly minimizing waste.

## Materials and methods

### Design and working principle of a two-stage injection jet mixer

**Fig 1:**
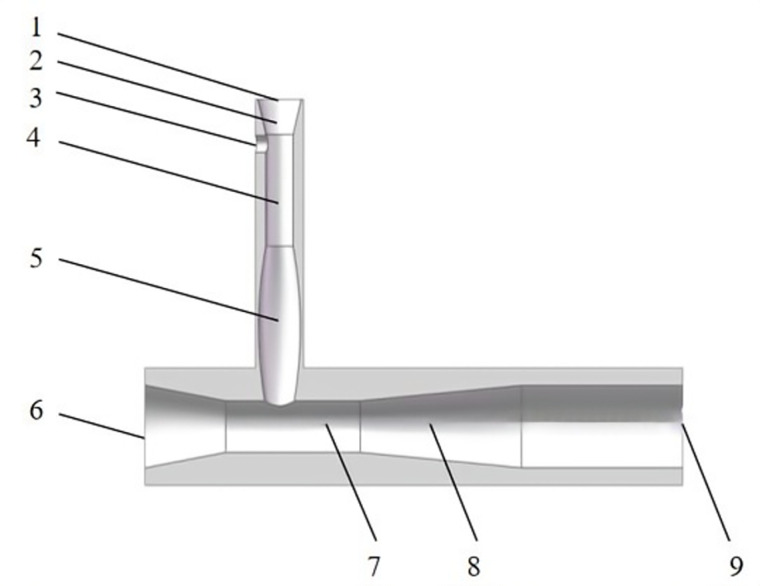
Structure of the two-stage injection jet on-line mixer. This figure shows a three-dimensional structural diagram of a two-stage injection jet in-line mixer, where each numerical sequence number corresponds to the structure name as follows: 1. First-stage water injection port 2. First-stage shrink tubing 3. First-stage pesticide injection ports 4. First-stage throat piping 5. First-stage diffusion shrink tubing 6. Second-stage water injection port 7. Second-stage throat piping 8. Second-stage diffusion tube 9. Outlet.

The structure of the two-stage injection jet mixer is shown in Fig 1, which consists of two independent single-stage jet mixers connected in series: the first-stage jet mixer and the second-stage jet mixer. The first-stage jet mixer mainly consists of a shrink tube, a throat pipe, a diffusion shrink tube, a water injection port and a pesticide injection port. The second-stage jet mixer mainly consists of a water injection port, a shrink tube, a throat pipe, a diffusion tube and a water outlet port. The mixers are manufactured using a cylindrical plexiglass material that is strong and resistant to corrosion, humidity and sunlight.

In a previous study on the actual space size of mixer loading, the diameter of the inlet of the primary jet mixer was 32 mm, and the diameter of the inlet of the secondary mixer was 65 mm [23]. The design and optimisation of the structural parameters of the mixer were carried out according to Burdock’s effort equation, the Reynolds equation, average flow velocity calculation data regarding the physical characteristics of two-phase liquids, and design criteria for Venturi tubes [28]. The numerical simulation and analysis of a two-stage injection jet mixer were conducted using ANSYS FLUENT 16.0, a commercial software for computational fluid dynamics (CFD) developed by ANSYS Inc. This approach examined the impact of various parameters, including nozzle diameter, throat length, and diffuser angle, on the performance of the mixer, as well as the influence of entrance velocities on uniformity.

This study investigates the performance of mixing water-soluble pesticides with water in a mixer for interpenetrating mixing of two-phase fluids, which is a fully turbulent model, and the flow field is selected as a standard K-ε turbulence model. Based on the flow velocity u at the throat of the mixer, the throat diameter D, the liquid viscosity coefficient μ and the liquid density ρ, the Reynolds number can be calculated:


$$\begin{array}{c} R_{e}=\frac{\rho uD}{\mu } \end{array}$$ (1)

So the mixture is solved utilizing the momentum equation, which describes its discrete phases through relative velocities. The mixing ratio is:


$$\begin{array}{c} \text{q}=\frac{\Delta Q_{s}}{\Delta Q}=\frac{\Delta Q_{s}}{Q_{1}+Q_{2}} \end{array}$$ (2)

Where $\Delta Q_{s}$ is the flow rate of drug solution, $Q_{1}$ is the flow rate of primary water, $Q_{2}$ is the flow rate of secondary water, and ΔQ is the total water volume, m^3^·s^-1^.

CFD simulations are performed for different mixing ratios to extract the flow velocity distributions and calculate the corresponding $R_{e}$ ranges to verify that the flow is in a sufficiently turbulent state. The turbulence intensity can be calculated by outputting the turbulent kinetic energy k versus the mean flow velocity u from the CFD simulation:


$$TI=\frac{\sqrt{\frac{2}{3}k}}{u}\times 100\;\%$$ (3)

Simulation experiments can be based on this to analyze the effect of regions of high turbulence intensity (e.g., throats and diffusion tubes) on mixing homogeneity.

**Fig 2:**
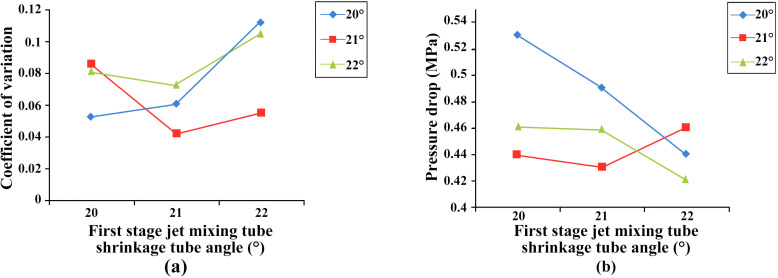
Influence of shrink tube angle on turbulent dissipation in the first stage of a mixer. The figure shows the coefficient of variation and pressure drop at the outlet face of the mixer for the first-stage mixer shrink tube angle and the second-stage mixer shrink tube angle of 20°, 21°, and 22°, respectively. Figure (a) shows the coefficient of variation for each combination of angles. Figure (b) shows the pressure drop for each angle combination.

**Fig 3:**
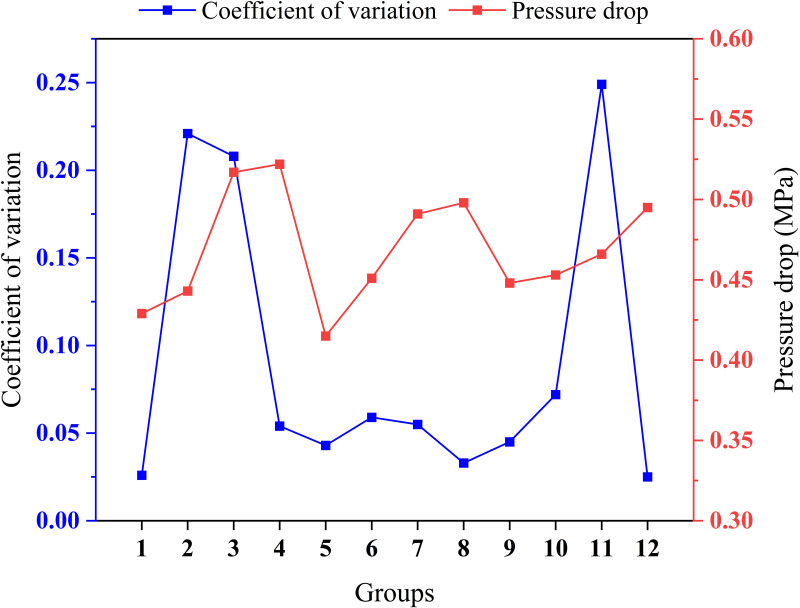
Effect of Mixer Nozzle Diameter on Turbulent Dissipation. The figure shows the coefficient of variation and pressure loss at the outlet face of the mixer for 12 combinations of parameters for the first stage jet nozzle diameters of 7 mm, 8 mm, and 9 mm, and for the second stage jet nozzle diameters of 16 mm, 18 mm, 20 mm, and 21 mm, respectively.

At the same time, the CFD simulation design method was utilized to investigate the effects of 12 combinations of nozzle diameters (three primary jet nozzle diameters and four secondary jet nozzle diameters) and three diffuser tube angles on turbulent dissipation, and the test reflected the effects of turbulent dissipation by two factors, namely, liquid coefficient of variation and pressure loss at the outlet surface of the mixer, and the results are shown in Figs 2 and 3. The four key structural parameters of the primary jet mixer throat length, the secondary jet mixer throat length, the primary jet spreading tube short axis length and the secondary jet diffusion tube angle are simulated by the four-factor, four-level orthogonal method to verify the mixing liquid coefficients of variation for the different combinations of parameters, Table 1 shows the orthogonal test factor level table, and the four-factor mean values are analyzed intuitively as shown in Table 2, where Ei is the mean value of the factors at each level. The results show that the optimal combination of factor levels is A_2_B_1_C_2_D_1_, i.e., the length of the primary throat is 45 mm, the length of the secondary throat is 80 mm, the length of the short axis of the primary jet diffuser tube is 26 mm, and the angle of the secondary jet diffuser tube is 7°. The influence of the four factors was B > D > A > C, i.e., the second-stage jet mixer throat length had the greatest influence, followed by the second-stage jet diffuser tube angle, the first-stage jet mixer throat length, and the first-stage jet spreader tube short-axis length had the least obvious influence on the test results. From the results of ANOVA as shown in Table 3, the significance results are consistent with the results of intuitive analysis. The optimal combination of structural parameters for the two-stage injection jet mixer was finalized by CFD simulation analysis, as shown in Table 4.

**Table 1:** Level table of orthogonal test factors.

Level	A	B	C	D
	Length of first stage jet mixer throat pipe (mm)	Length of second stage jet mixer throat pipe (mm)	Short axis length of first stage jet diffusion shrinkage tube (mm)	Angle of second stage jet diffuser tube (°)
**1**	40	80	32	7
**2**	45	105	26	9
**3**	54	126	20	11
4	63	147	14	13

**Table 2:** Orthogonal design scheme and intuitive analysis.

Serial number	Level of relevant factors				Combination	Simulation results
	A	B	C	D		
**1**	1	1	1	1	A_1_B_1_C_1_D_1_	0.021
**2**	1	2	2	2	A_1_B_2_C_2_D_2_	0.114
**3**	1	3	3	3	A_1_B_3_C_3_D_3_	0.115
**4**	1	4	4	4	A_1_B_4_C_4_D_4_	0.078
**5**	2	1	2	3	A_2_B_1_C_2_D_3_	0.020
**6**	2	2	1	4	A_2_B_2_C_1_D_4_	0.186
**7**	2	3	4	1	A_2_B_3_C_4_D_1_	0.032
**8**	2	4	3	2	A_2_B_4_C_3_D_2_	0.024
**9**	3	1	3	4	A_3_B_1_C_3_D_4_	0.125
**10**	3	2	4	3	A_3_B_2_C_4_D_3_	0.431
**11**	3	3	1	2	A_3_B_3_C_1_D_2_	0.092
**12**	3	4	2	1	A_3_B_4_C_2_D_1_	0.055
**13**	4	1	4	2	A_4_B_1_C_4_D_2_	0.024
**14**	4	2	3	1	A_4_B_2_C_3_D_1_	0.031
**15**	4	3	2	4	A_4_B_3_C_2_D_4_	0.095
**16**	4	4	1	3	A_4_B_4_C_1_D_3_	0.125
**E1**	0.082	0.048	0.106	0.035		
**E2**	0.066	0.191	0.071	0.064		
**E3**	0.176	0.084	0.074	0.173		
**E4**	0.069	0.071	0.141	0.121		

**Table 3:** ANOVA.

Source	Degrees of freedom	Sum of squared deviations	Mean square	Adjust the mean square	Extremely poor
**A**	3	0.032851	0.032851	0.010950	2.14
**B**	3	0.048292	0.048292	0.016097	3.14
**C**	3	0.013006	0.013006	0.004335	0.85
**D**	3	0.045229	0.045229	0.015076	2.94
**Error**	3	0.015360	0.015360	0.005120	
**Total**	15	0.154740			

**Table 4:** Key structural parameters of the two-stage injection jet on-line mixer.

Structure of the mixer	Parameter values
**First-stage jet nozzle diameter**	9 mm
**Second-stage jet nozzle diameter**	21 mm
**First-stage throat length**	45 mm
**Second-stage throat length**	80 mm
**Short-axis length of the primary expansion pipe**	25 mm
**Angle of the secondary diffusion tube**	7°
**Angle of the contraction tube**	21°

The working principle of the two-stage injection jet mixer is as follows. First, the high-pressure water passes through the conical pipe in the first-stage jet mixer, and pressure energy is converted to kinetic energy by accelerating the fluid flow. This high-velocity fluid reaches the injected pesticides in the throat, resulting in molecular reactions [29]. The Brownian motion of the molecules causes them to diffuse from higher concentrations to lower concentrations before flowing into the shrinkage tube. During this process, velocity energy is converted into pressure energy and then again into velocity energy. The mixed solution then enters the secondary mixer as a high-velocity fluid, where it is again mixed with water for dilution [30].

### Test equipment and materials

**Fig 4:**
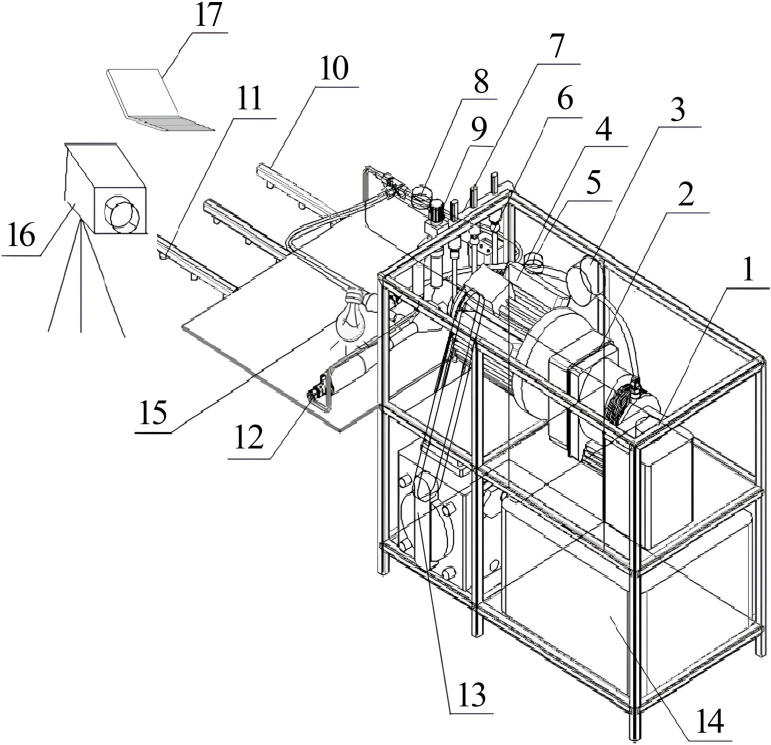
Two-stage injection jet mixer test system. This figure shows the composition of a two-stage jet mixer mixing test system and how it is combined, where each numerical serial number corresponds to the name of the component as follows: 1. Pesticide tank 2. Pesticide pump 3. Pesticide flow meter 4. Motor 5,8. Pressure gauge 6. Proportional valve 7. Water divider 9. Water flow meter 10. Spray bar 11. Nozzle 12. Mixer 13. Diaphragm pump 14. Water tank 15. Violet light 16. High speed camera 17. Computer.

The two-stage injection jet mixer test system consists of a water inlet unit, a pesticide inlet unit, a mixer unit, a spray unit and a high-definition photography unit, the test stand is shown in Fig 4. The water inlet unit used was a YG-4160 piston diaphragm pump from Yonggao Company, with a rotational speed of 600 rpm, a power match of 5.5 kW, a flow rate of 160 L·min^-1^, and a pressure of 3 MPa. The external power for the pump is provided by a YE2-160M2-8 three-phase asynchronous motor from Zhejiang Zhonglong Electric Motor Co., Ltd., which yields 5.5 kW of power and a voltage of 380 V. The water from the diaphragm pump outlet is connected to the inlet end of the mixer through a tee, and all of them use YF-DN40-S turbine flowmeter from Guangdong Dijiang Sensor Company to detect the flow rate, which is in the range of 2 ~ 150 L·min^-1^, with an accuracy of ±0.5%. The 05 type electric proportional valve is selected according to the requisite flow demand. For the pesticide inlet unit, a DM-33–03-GX new Dautz diaphragm metering pump from Beijing Ark Tongda Electromechanical Technology Co., Ltd., was used with a flow rate of 1 ~ 30 L·h^-1^, a voltage of 220 V, and an accuracy error of ±2%. In consideration of the requisite working pressure, the DP-160 booster pump has been selected for the purpose of pressurising the pesticides accurately conveyed by the metering pump in real time. The booster pump operates at a working voltage of DC24V, with a maximum pressure of 1 MPa and a maximum flow rate of 7 L·min^-1^. The pesticide flow metre used was a float flow metre from Jiangsu Anquan Instrument Co. The water inlet and outlet of the mixer included Y-60 ordinary pressure gauges from Red Flag Instrumentation Co., Ltd., with pressures ranging from 0 ~ 1.6 MPa. The spray nozzle used was a VP110015 from Fengnuo Company, with a pressure of 0.2 MPa and a flow rate of 0.45 L·min^-1^. The high-definition photography unit includes a high-definition camera, a mobile light source, an image capture card and a mobile workstation. The mobile light 5 used was a 50-W domestic LED violet light, and the image integration card used was pcoCamware3. The high-definition camera is capable of capturing high-resolution images with secondary composition capability. It is able to transfer these images to the data acquisition card via the data interface, where they can be analysed using the mobile workstation.

### Mixer qualitative test

#### Purpose of the test.

To ensure that the mixing effect of the developed system was sufficient for pesticide application, a test platform was built in the laboratory to analyse the distribution of pesticides in the two-stage injected jet mixer using high-speed photography. This qualitative test was performed to ensure that the pesticides and water did not separate and that no other issues occurred during mixing. Moreover, it was verified that a given amount of pesticide in the tests could meet the measured test amount in the quantitative test, which is in line with the relevant standard.

The image analysis method employs the optical reaction of the image to analyse it digitally in a linear operation. This method offers several advantages, including non-contact operation, online monitoring, flexibility, ease of integration, and more. It can rapidly, accurately, and safely detect the mixing effect of a solution. The image analysis method is based on the use of MATLAB software to analyse images and perform data processing. Pixel index plots in the axial direction, pixel index plots in the radial direction, and root mean square error plots of the solution inside the mixer were obtained through the application of statistical methods [7,31].

A water-soluble, yellow-green fluorescent powder produced by Caihui Chemical Co Ltd was selected as the pesticide tracer for the forthcoming test. And to maintain consistent image acquisition conditions, a violet light lamp with a wavelength equal to that of the tracer was chosen to ensure that stable and clear images were obtained.

#### Precalibration test.

**Fig 5:**
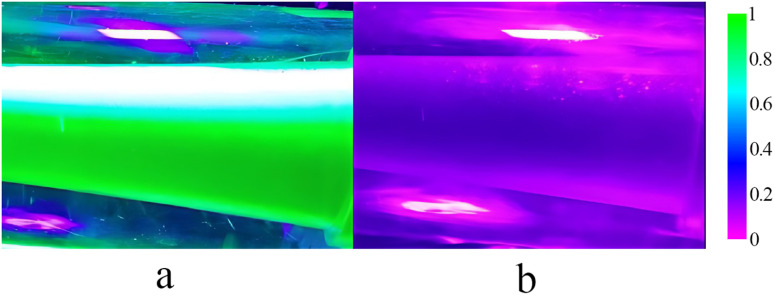
Original image captured by the camera. This figure shows the camera taking an image of the solution in the mixer, where figure (a) is pesticide solution image and figure (b) is pure water image.

The test water supply pressure is 0.1 MPa, water supply flow rate flow rate of 140 L·min^-1^. Based on the distance between the camera and a given object, the aperture and focal length were adjusted to maximise image clarity. To ensure the stability of image acquisition and a consistent frequency, the camera parameters must be consistently set and maintained. For the pure water calibration test, the pesticide pump was closed when the water pump was opened. This water then filled the entire mixer, and an image of the diffusion tube portion of the mixer was taken as a calibration aqueous solution. The water pump was closed during the pure water discharge process and then opened the pesticide pump when all the water is discharged. Then, the pesticide solution was added to the mixer. The imaging method described above was used for the pesticide solution for calibration. As image acquisition is a noncontact process and the mixer uses cylindrical plexiglass material, when the light illuminates the curved glass, refraction occurs, leading to the potential loss of some information and the distortion of pixels in the images. The upper part of the image in Fig 5(A) is more distorted than the other parts due to the direct light from the violet-light lamp, and the image in Fig 5(B) of pure water is less distorted. To mitigate pixel interference and the unfavourable effects of other factors during the tests, it was necessary to preprocess the collected images. Two images are cut into photos with the same pixel, and the subtraction operation in software analysis is used to perform the image difference. Through such subtraction operation, the same background or interference information in the two images can be removed, and the difference between the two images can be highlighted to obtain the interference-removed image, which is more conducive to further analysis and processing of specific targets or features in the image.

### Test method

First, the device connected to the high-speed camera, fixed tripod and camera is opened to ensure that the physical and camera levels are met. In accordance with individual experimental needs, the aperture and focal length are adjusted, and the high-speed camera system properties are set, such as the resolution, frame rate and trigger mode. The camera acquisition parameters are all set up to shoot images and save the file. Use the camera to take 20 photos in a row and choose the one with the best effect and the clearest image. The captured image is converted into JPG format and put into MATLAB for image and pixel data analysis. To avoid other negative factors in the image analysis, the image needs to be removed from the background and denoising process. The images are then normalized to remove the brightness differences due to the mixing ratio and retain the true concentration signal. The threshold of the region of interest is further distinguished, and then binariation is carried out. The threshold is chosen as 0.3 to realise axial and radial pixel analysis and root mean square error analysis in the same axis or same diameter direction. In digital image processing, thresholding and binarization are common methods for image segmentation and feature extraction. By setting an appropriate threshold, the pixels in the image are divided into foreground and background, and the image is binarized, which is convenient for subsequent operations such as axial and radial pixel analysis and root mean square error analysis in a specific region (area of interest) [7,32]. Ten experiments were conducted using this methodology, and data from faulty equipment or blurred images were excluded. Ultimately, eight valid datasets were retained for image processing and analysis. Following this, the mean of pixel index values was calculated for axial and radial pixel analysis. The root mean square error (RMSE) of pixel values in the region of interest for each image was subsequently determined, and the average of these RMSE values was taken as the final result for analysis.

### Quantitative test of the mixer

#### Test design.

Mixer performance was tested using cochineal instead of water-soluble pesticides, and UV spectrophotometry was used for quantitative analysis [33–35]. The four 6 g·L-1 cochineal solutions were diluted at a ratio of 3000:1 ~ 300:1 to obtain four standard samples of different concentrations. The UV spectrophotometer was set to an absorption wavelength of 507 nm [36], and the absorbance of the samples was measured sequentially, with the average value taken as the experimental data. Based on the concentrations of the standard samples and their corresponding absorbance values, a linear relationship was fitted as follows:


$$\begin{array}{c} \text{ABS}=10.5721\text{C}+0.01638 \end{array}$$ (4)

where ABS is the absorbance and C is the concentration of the pesticide solution in mg·mL^-1^.

In this paper, the concentration of the solution was calculated by determining its absorbance, and the relative error in the concentration of the mixed solution was derived by comparing the actual mass concentration of the sample solution with the expected mass concentration to assess the homogeneity of the pesticide-water mixture. Calculate the coefficient of variation of the homogeneity of the mixture according to the following formula, and judge the homogeneity of the mixture according to the magnitude of the coefficient of variation.


$$\begin{array}{c} \text{CV}=\frac{\xi }{\tau } \end{array}$$ (5)

where ξ represents the standard deviation of the mixer liquid concentration in g·L^-1^ and the actual mean value of the mixer liquid concentration in g·L^-1^.

#### Test method and steps.

The mixing uniformity of the mixer was first analyzed at different water flow rates by varying the water flow rate at each stage or by varying the total water volume of the diaphragm pump at different mixing ratios. Eight more tests were done for each group to obtain the coefficient of variation of uniformity.

Then the moment uniformity of the mixer was analyzed, and the flow rate of the water inlet unit was set to the maximum flow rate of 140 L·min^-1^ for the design demand, and the pesticides inlet unit was supplied according to a mixing ratio of 300:1 ~ 3000:1. A total of ten sampling points were selected within the plant protection spraying system. These included the outlet end of the mixer, the three outlet ends of the distributor, the first nozzle and the end nozzle of the three-way spray bar. A measuring cup was employed to collect the sample solution. Each sampling period was 2s, and the sampling interval was 30s. Each group is sampled five times. A Shimadzu UVmini-1240 ultraviolet spectrophotometer was used to determine the absorbance of the samples, and the average was used in subsequent analyses. The absorbance accuracy of the UV spectrophotometer is ± 0.002 Abs. The temporal homogeneity of the average values over 5 intervals and the spatial homogeneity of the 10 samples were investigated to assess the continuous mixing ability of the two-stage jet mixing system.

The specific steps of the test were as follows.

(1) At the outlet end of the mixer, after the mixing system was normally operated for one minute at the preset mixing ratio and the pipelines were operating normally, the mixing solution was sampled for a fixed length of time, with a given interval between samples. The temporal uniformity of mixing at the outlet end of the mixer was then assessed by analysing samples of the collected solutions.
(2) In accordance with the preset mixing ratio, the mixing system is typically initiated and operated for a duration of one minute. In the context of normal pipeline operation, sample solutions are collected from 10 sampling points at regular intervals. The absorbance of the sample solution was analysed in order to determine the concentration of the mixing solution flowing from the mixer to the manifold, pipeline and individual spray nozzles. This was done as a means of verifying the spatial distribution uniformity of the mixer and the supporting spray system.

In accordance with the aforementioned test method, ten sets of tests were repeated for all groups with different mixing ratios. The average of the sample concentrations measured in the ten tests was then taken for data analysis in order to reduce the chance of an erroneous result.

## Results

### Analysis of the qualitative results of the two-stage jet mixer

#### Mixer axial and radial pixel analysis.

In the test, the experimental images were compared with the calibration images, and the uniformity of mixing was analysed based on the axial or radial distribution of the solution in the images. The axial and radial directions of the domain of interest were set, 8 regions were selected at equal spacings from top to bottom in the axial region, and the intervals, with 10 corresponding pixel points in the axial direction, were denoted with different colours. Additionally, 6 regions with equal spacing were selected from left to right in the radial region, and the intervals, with 20 corresponding pixel points in the radial direction, are denoted with different colours.

**Fig 6:**
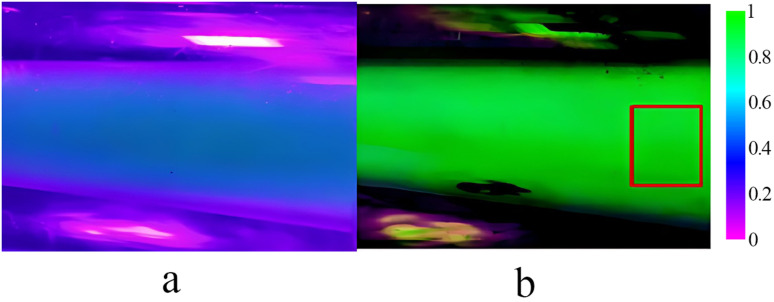
Images of a mixed solution based on 300-fold dilution. This figure shows an image of a pure pesticide that has been diluted 300 times and captured in a mixer. Figure (a) shows the original image and figure (b) shows the pre-processed image with the location of the region of interest labelled.

Fig 5(a) presents a control image of the pure pesticide. A 6 g·L^-1^ phosphor solution was prepared on the test bench for 300-fold dilution and mixing, and images of the mixing process were captured, as shown in Fig 6(a). The acquired images were input into MATLAB software for background removal and denoising, and the domain of interest was selected, as shown in Fig 6(b).

**Fig 7:**
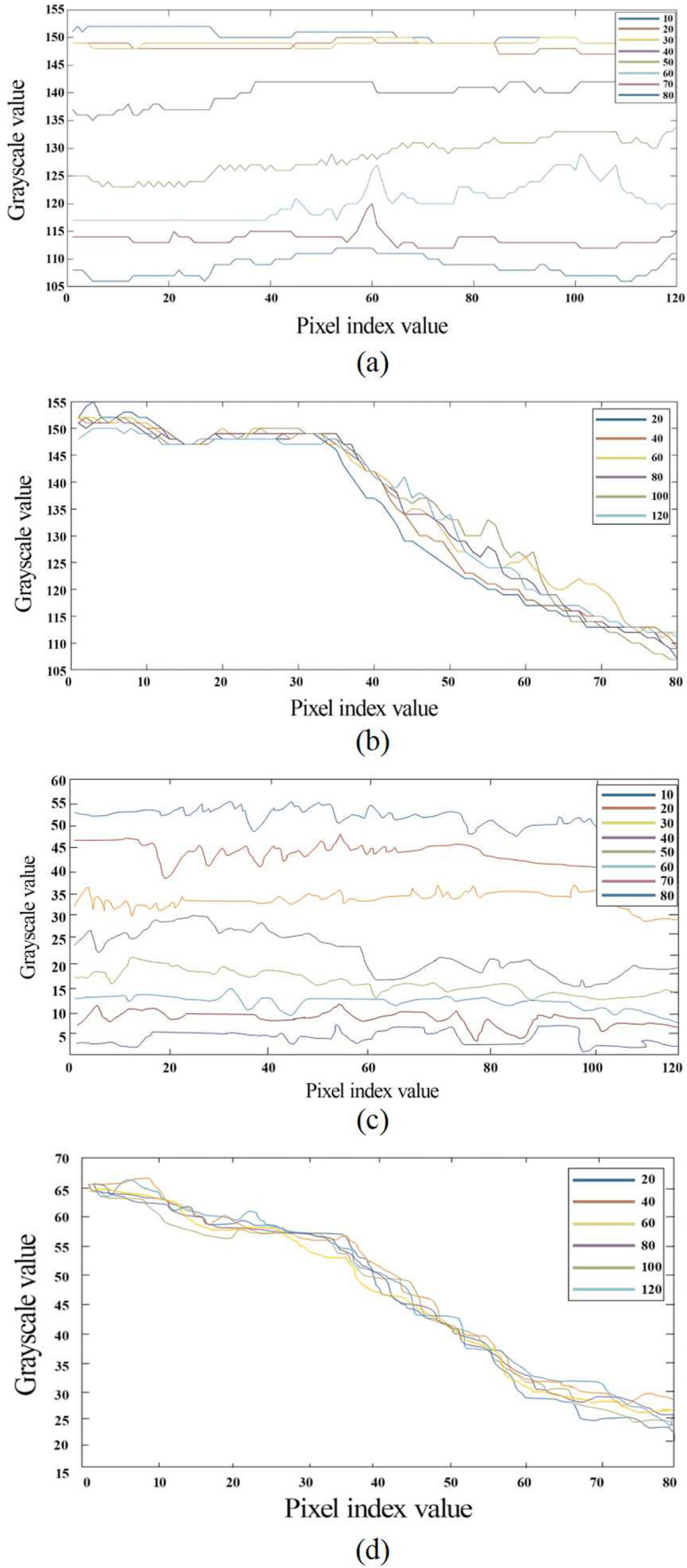
Pixel index distribution. This figure shows equally spaced radial and axial pixel profiles of processed images of pure pesticide solutions and 300:1 images of mixtures of water and pesticide, with lines of different colours representing pixel points within a region of interest selected at equal intervals according to a certain direction. Figure (a) is plot of the pixel index and grayscale values for pure pesticides in the axial direction, figure (b) is plot of the pixel index and grayscale values for pure pesticides in the radial direction, figure (c) is plot of the pixel index and grayscale values in the axial direction for a pesticide mixed at a 300:1 ratio with water, and figure (d) is plots of the pixel index and grayscale values in the radial direction for a pesticide mixed at a 300:1 ratio with water.

The test results obtained for the pixel index distribution are shown in Fig 7, where the horizontal coordinate is the pixel index value (the positional identification of each pixel point in the image) and the vertical coordinate is the grayscale value (the brightness of each pixel point in the image). According to the results in Fig 7(a) and (b), the fluctuations in the pesticide distribution in the axial direction are not large, but in the radial direction, the grey value is greater in the upper part of the mixer than in the lower part. This is because the violet light lamp was installed on the upper side of the mixer and enhanced the local imaging result. As shown in Fig 7(c) and (d), each pixel-scale curve tends to be stable and fluctuates little in the axial direction, while in the radial direction, there is a slow decreasing trend, with little fluctuation between the distribution curves. These plots are consistent with the axial and radial curves of the pesticides in Fig 7(a) and (b), indicating that for a 300:1 ratio of water to pesticide, uniform mixing in the axial and radial directions is achieved with the two-stage jet mixer.

#### Root-mean-square error analysis of the mixer.

In order to investigate the stability and applicability of the mixer in question, the RMSE of the mixture images at different mixing ratios were compared and the mixing uniformity was analysed based upon image analysis.

**Fig 8:**
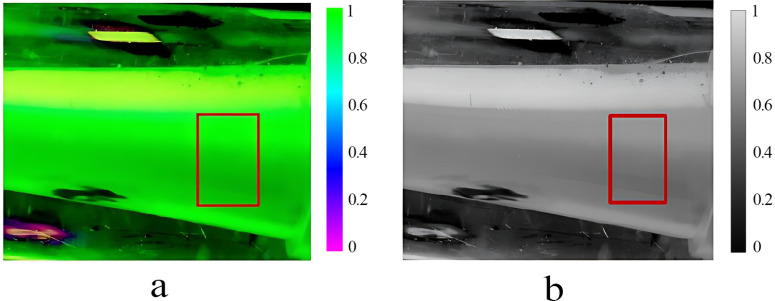
MATLAB AOI selection for an image of a mixed solution with 300-fold dilution. This figure shows Matlab’s selected regions of interest for the image of a pre-processed 300-fold dilution of the solution. Figure (a) is selection of the AOI after background and Figure (b) is Greyscale image denoting the AOI removal and denoising.

**Fig 9:**
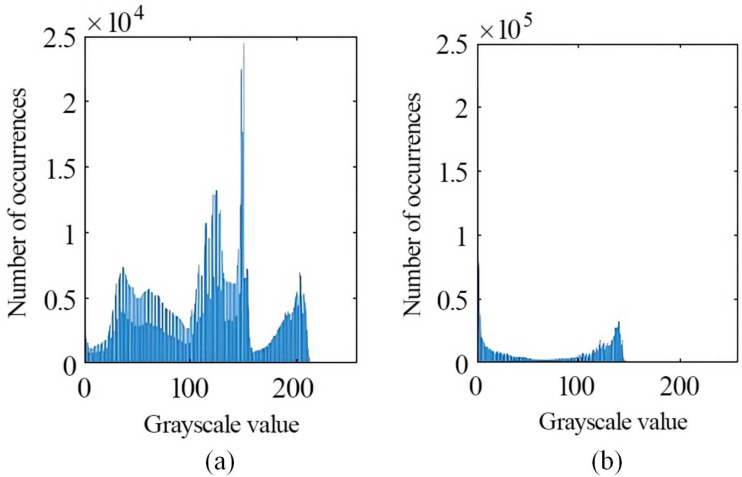
Histogram of greyscale. This figure shows the grey scale histograms of the pure pesticide and the 300-fold diluted solution. Figure (a) is calibrated pure pesticide solutions and figure (b) is mixed solution with 300-fold dilution.


The image of the liquid mixing and flowing within the mixer has been imported into MATLAB software for subsequent analysis. This was accomplished via the imread function. The length and width of each image and the pixel value were maintained as constants. The back end of the mixer diffusion tube was designated as the area of interest (AOI). The rectangle ‘Position’ (261, 150, 80, 120) should be defined in accordance with the dimensions of the selected area. The AOI was established as AOI = Pic0 (150:229, 261:380), and the pixel value was 120*80, as shown in Fig 8(a). Fig 8(b) shows the grey scale image of the region of interest. The total AOI domain can be obtained by this method. Pixels from the AOI were processed by using a binarization method with a threshold value of 0.3. The points with pixel values greater than 0.3 were assumed to be associated with fluorescent particles, and those with pixel values less than 0.3 were assumed to be associated with aqueous solution. With Equation (2), the mixing ratio of pesticide to water in the mixer was calculated. ROI_gray = rgb2gray (ROI) was defined to obtain the grey value of the AOI, and hist_gray = imhist (ROI_gray) was defined to obtain the grey histogram of different parts of the AOI. As shown in Fig 9(a), the peak of the histogram of the pure pesticide solution coincides with the middle of the image and spans a wide range of nonzero-value regions; the maximum value is 2.5*104, indicating high contrast. As shown in Fig 9(b), the peak of the histogram of the image of the 300-fold diluted mixed solution coincides with the left side of the image, which indicates that the image is darker and lighter in colour.

$$\begin{array}{c} \mu =\rho /\tau \end{array}$$ (6)

where ρ is the area occupied by the fluorescent particles and τ is the total area of the selected AOI in the mixer.

The root mean square error (RMSE), also known as the standard value error, is defined as the square root of the mean of the squared differences between the acquired image test data and the standard data value. This error is calculated by taking the square root of the mean of the squared differences between the sum of the squares and the number of times the number of tests is compared to the n times. The level of the results can be opened by following the steps outlined in Equation (7). Over multiple trials, the best calibration image was used, and the relatively uniform region of the solution near the diffusion port was selected as the AOI. The RMSE can be used to assess the degree of dispersion of fluorescent particles in the AOI, as it reflects large differences well and is thus an effective evaluation metric.


$$\begin{array}{c} \text{RMSE}=\sqrt[{2}]{\frac{{\left(a_{1-}\forall \right)}^{2}+{\left(a_{2-}\forall \right)}^{2}+{\left(a_{3-}\forall \right)}^{2}\ldots {\left(a_{n-}\forall \right)}^{2}}{n}} \end{array}$$ (7)

where a1, a2, a3... and an are the trial measurement pixel values; ∀represents the average of n groups of trial pixels; n is the number of trials; and RMSE is the root mean square error.

**Fig 10:**
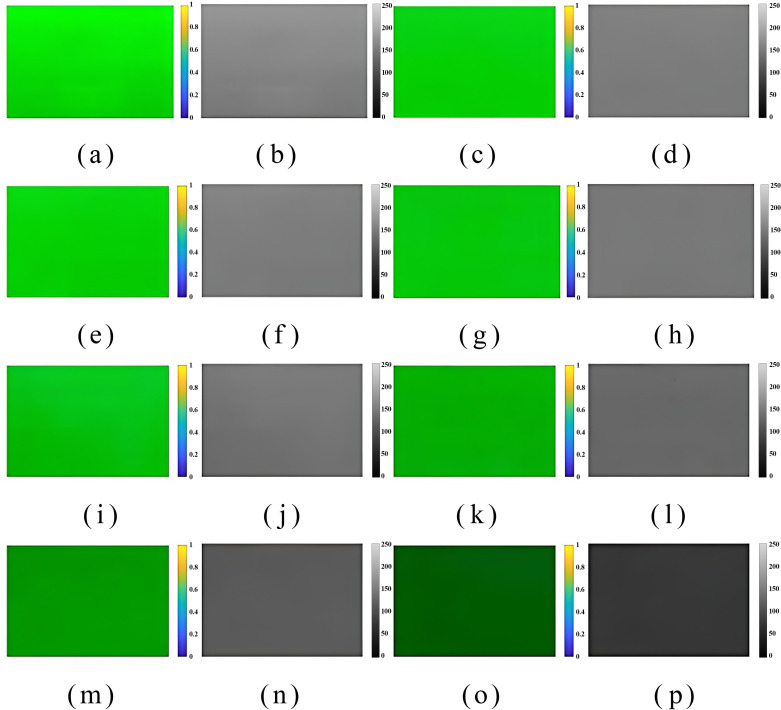
Colour distribution in the area of interest for different solution mixing ratios. This figure shows the images of the region of interest of the images of mixed solutions of pure pesticide, water and pesticide mixed in the ratios of 300:1, 500:1, 1000:1, 1500:1, 2000:1, 2500:1, 3000:1 and their grey scale maps for comparison. Notes: (a) Area of interest of the calibrated physical solution (b) Grayscale diagram of the area of interest of the calibrated physical solution (c) Area of interest diagram of the physical solution with 300-fold dilution (d) Grayscale diagram of the area of interest of the physical solution with 300-fold dilution (e) Area of interest diagram of the physical solution with 500-fold dilution (f) Grayscale diagram of the area of interest of the physical solution with 500-fold dilution (g) Area of interest diagram of the physical solution with 1000-fold dilution (h) Grayscale map of the area of interest of the physical solution with 1000-fold dilution (i) Grayscale map of the area of interest of the physical solution with 1500-fold dilution (j) Grayscale map of the area of interest of the physical solution with 1500-fold dilution (k) Grayscale map of the area of interest of the physical solution with 2000-fold dilution (m) Grayscale map of the area of interest of the physical solution with 2500-fold dilution (n) Grayscale map of the area of interest of the physical solution. (o) Map of the physical solution domain of the area of interest for the solution with 3000-fold dilution (p) Map of the physical solution domain of the area of interest for the solution with 3000-fold dilution.

**Fig 11:**
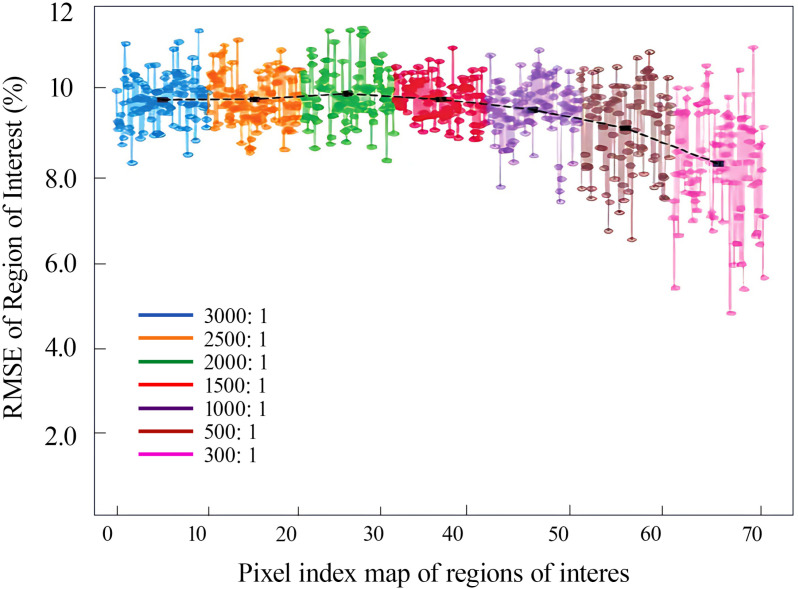
RMSE of the AOI for water-pesticide mixing ratios ranging from 300:1 to 3000:1. This figure shows the percentage of root mean square error of pixel values in the region of interest of the mixed solution image for water and pesticide mixing ratios of 300:1, 500:1, 1000:1, 1500:1, 2000:1, 2500:1, and 3000:1. Different coloured lines indicate different mixing ratios, and the black curve is the mean curve fitted to the root mean square error percentage of the solution for each mixing ratio.

After denoising and background removal of the pure pesticide and mixed solution at a mixing ratio of 300:1 ~ 3000:1, the AOI and the greyscale image of the AOI were input into the analysis software, and the resulting colour distribution bar charts are displayed in Fig 10. The larger the mixing ratio was, the brighter the image, and as the mixing ratio decreased, the images displayed darker and lower grey values. Based on data processing and Equation (7), the maximum pixel value in the AOI image of the pure pesticide was found to be 106, the minimum pixel value was 153, the mean value was 38.75, and the root mean square error was 1.5228. According to Equation (8), the RMSE was 3.24%. As described, various information was input into MATLAB, and the percentage of pixels with nonuniform mixing of water and pesticides was determined, as shown in Fig 11. As the mixing ratio and solution concentration in Fig 10 increase, the corresponding pixel index value also rises. When the water-to-pesticide mixing ratios were 300:1, 500:1, 1000:1, 1500:1, 2000:1, 2500:1 and 3000:1, the percentage RMSEs were 8.57%, 9.64%, 9.82%, 9.86%, 9.94%, 9.85% and 9.87%, respectively. The analysis of Fig 13 indicates that as the mixing ratio increased, the root mean square error percentage overall shows a increasing trend by a small margin. However, the change is minimal, and the overall state remains stable, with the maximum difference observed at only 1.37%. The mixing ratio of water and pesticide was 300:1, with minimum errors of 8.57%, and 2000:1, with a maximum error of 9.94%. Overall, the root mean square error percentages of the different mixing ratios are found to be within a stable interval, indicating that the solutions of each ratio have a similar mixing effect and good homogeneity. Compared to the previous study by Chen [7], the RMSE of the mixer designed in this research has been reduced by 33.06%. The presence of a minor root mean square error in the mixture suggests that the solution can be mixed to a more homogeneous state. However, it is unable to achieve complete homogeneous mixing, which is attributable to external factors such as systematic error. Such occurrences are also permitted. In particular, when the concentration of the mixture is low and the pesticide content is minimal, the actual mixing effect is likely to be insignificant. However, the subsequent flow of the pipeline and the vibration of the plantation machine during operation may facilitate further mixing of pesticides and water.

**Fig 12:**
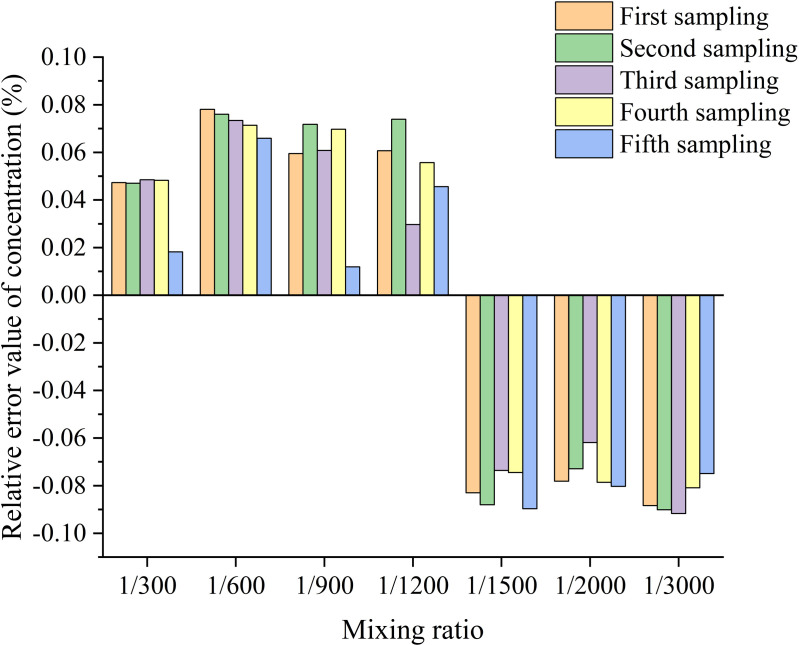
Plot of the relative error variation in the temporal distribution of the sample concentration of the mixture. This graph shows the relative magnitude of error in the mass concentration of a mixture of water and pesticide at ratios of 300:1, 500:1, 1000:1, 1500:1, 2000:1, 2500:1, and 3000:1. The different coloured bars indicate samples taken at different times.

**Fig 13:**
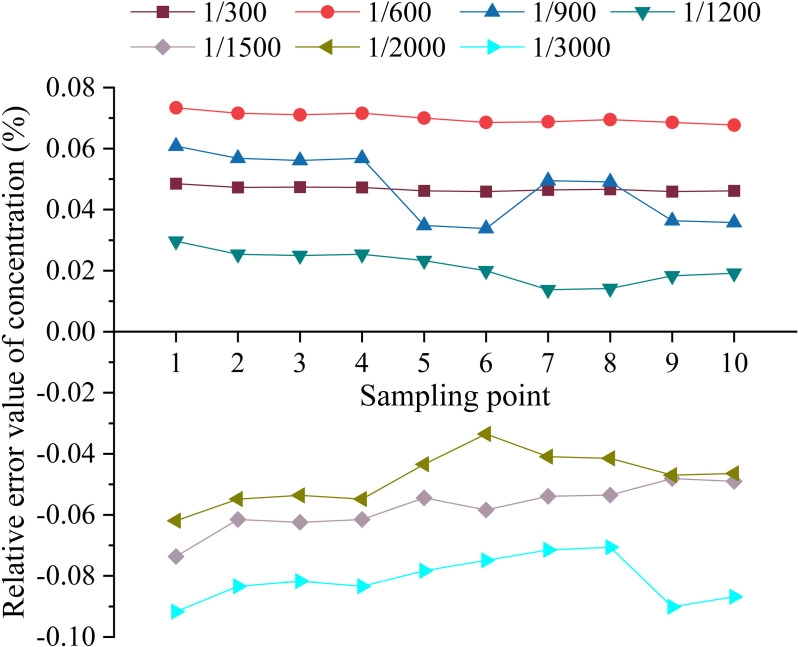
Plot of relative error variation in the spatial distribution of sample concentrations of the mixture. This figure shows the relative mass concentration of a mixture of water and pesticide compared at different sampling points. The different coloured curves indicate different mixing ratios.


$$\begin{array}{c} \delta =\frac{\text{RMSE}}{\left(\tau_{A}-\tau_{a}\right)}*100\\ \% \end{array}$$ (8)

where δ represents the RMSE as a percentage, τA represents the maximum pixel value, τa represents the minimum pixel value, and RMSE represents the root mean square error.

By analyzing the experimental image data, the SNR values at different mixing ratios were calculated and compared with the RMSE results, as shown in Table 5.

**Table 5:** Comparison of SNR and RMSE results for solutions with different mixing ratios.

Mixing ratio	SNR (dB)	RMSE (%)	Signal strength (gray scale average)	Noise standard deviation (gray value)
**300: 1**	34.2	8.57	152	8.3
**500: 1**	32.1	9.64	128	8.5
**1000: 1**	28.7	9.82	98	8.7
**1500: 1**	26.5	9.86	82	8.9
**2000: 1**	24.8	9.94	71	9.1
**3000: 1**	22.3	9.87	60	9.3

From the above table, it can be seen that as the dilution increases (mixing ratio from 300:1–3000:1), the pesticide concentration decreases and the signal strength (gray scale mean) diminishes while the background noise standard deviation slightly increases, resulting in a decrease in the SNR from 34.2 dB to 22.3 dB.The RMSE shows a small upward trend as the SNR decreases (8.57% → 9.94%), indicating that the noise has a greater impact on the low concentration homogeneity assessment of the mixture increased.

The results show that at high dilutions (e.g., 3000:1), the fluorescent particles are sparse and the signal intensity is close to the noise level (SNR < 25 dB), resulting in the fluctuation of the gray value being amplified by the noise and the deviation of the RMSE calculation being increased. At high dilution (e.g., 3000:1), the fluorescent particles are sparse and the signal intensity is close to the noise level (SNR < 25 dB), resulting in the fluctuation of the grayscale value being amplified by the noise and the deviation of the RMSE calculation increasing. The reason may be that despite the reduction of stationary noise by background deduction and threshold binarization (threshold 0.3), edge pixel distortion due to curved refraction of the cylindrical Plexiglas container and dynamic noise generated by the top of the mixer close to the UV source accounted for a significant proportion at low SNR.

### Quantitative analysis of the two-stage jet mixer

The uniformity of the mixture after mixing the liquid inside the mixer should not only consider whether the uniformity of the liquid mixing changes with time but also consider whether the flow of the mixed liquid in the pipeline of the spraying system will cause the uniformity of the liquid to change. In this regard, this paper analyses the uniformity of the mixture from the two aspects of temporal distribution and spatial distribution. To verify the applicability of the mixer, whether it meets the requirements of plant protection work for a variety of crops must be determined.

#### Mixing uniformity analysis of mixer at different flow rates.

In the previous study [23], the mixing uniformity of the solution at different drug flow rates and different water flow rates were tested respectively, and the results showed that the mixing coefficient of variation of each group of tests was lower than 5%, and the mixing uniformity could be achieved, which was due to the internal structure of the mixer meets the requirements of uniform mixing of the drug and water and the two-stage injection mixer can achieve the same mixing effect in a wide range of mixing ratios. From this analysis, it can be seen that no matter changing the water flow rate or drug flow rate, it can be proved that the two-stage injection jet mixer meets the design requirements. Here, the experimental study of mixing uniformity under different pressure conditions is carried out in this paper, and the results are shown in Table 6.

**Table 6:** Coefficient of variation under different pressure conditions.

Mixing ratio	Mean mass concentration (mg·L^-1^)		Standard deviation (mg·L^-1^)		Coefficient of variation (%)	
	0.1MPa	1MPa	0.1 MPa	1 MPa	0.1 MPa	1 MPa
**1:300**	10.729	10.44	0.347	0.251	5.63	5.658
**1:600**	5.291	5.358	0.203	0.124	6.136	5.52
**1:900**	3.577	3.761	0.169	0.1	7.119	5.858
**1:1000**	3.198	3.436	0.141	0.117	6.787	6.749
**1:1500**	2.277	2.395	0.078	0.082	5.653	6.655
**1:2000**	1.826	1.791	0.102	0.07	8.274	7.203
**1:3000**	1.274	1.315	0.082	0.035	9.17	5.604

As can be seen from the data in the table, under atmospheric conditions (0.1MPa), the coefficient of variation of mixing uniformity of different concentrations of the mixture basically meets the design requirements, of which the maximum value of 9.17% for the mixing ratio of 1:3000, the minimum value of 5.63% for the mixing ratio of 1:300, and the coefficient of variation of the mixing uniformity will be with the mixing ratio increases and increases. The most important reason for this phenomenon is that during the test, if the mixing ratio increases, the flow rate of the liquid should be reduced, resulting in an increase in the internal pressure of the mixer, and the liquid can neither enter the mixer according to the pre-set requirements. Under pressurized conditions (1MPa), the coefficient of variation of mixing uniformity of the pesticide on-line mixing system is less than 8%, which indicates that under pressurized conditions, the mixing performance of the system is more stable, no matter whether it is a high-concentration mixing solution or a low-concentration mixing solution is able to be mixed stably.

#### Analysis of the temporal uniformity of mixing.

In order to investigate the relationship between the mixing performance of the mixer and the mixing time, and to verify whether the mixing performance of the mixer will continue to be stable under continuous operation, the homogeneity of the time distribution of the mixture flowing out of the outlet end of the mixer was analysed.

For the mixing test platform, the mixing ratio of pesticides and water was changed at the outlet end of the mixer in accordance with the quantitative test program. [1] Samples were collected 5 times. After sampling, each sample was placed in a spectrophotometer for real-time detection to determine the sample concentration at different mixing ratios, as shown in Table 7. The sample concentration was expressed in mg·L^-1^. The relative errors of the samples are shown in Fig 12.

**Table 7:** Actual and expected concentrations of samples sampled at different times for each mixing ratio.

Mixing ratio	First sampling	Second sampling	Third sampling	Fourth sampling	Fifth sampling	Expected concentration	CV (%)
**300:1**	9.548	9.551	9.537	9.539	9.821	10	1.29
**600:1**	4.638	4.647	4.658	4.667	4.691	5	0.44
**900:1**	3.143	3.107	3.139	3.113	3.291	3.33	1.98
**1200:1**	2.357	2.328	2.428	2.368	2.391	2.5	1.58
**1500:1**	2.181	2.193	2.159	2.161	2.197	2	0.36
**2000:1**	1.627	1.618	1.599	1.628	1.631	1.5	0.80
**3000:1**	1.097	1.099	1.101	1.088	1.081	1	0.77

As shown in Table 7, the difference between the actual concentration of the pesticide-water mixture collected after actual mixing in the two-stage injection jet mixer and the expected concentration is very small, which indicates that the mixing of pesticide and water is highly homogeneous. Additionally, the error between the concentrations of the solutions sampled at multiple intervals and the same mixing ratio is very small, which further suggests that the mixing performance is stable and that the temporal distribution of the mixture is uniform. There are some differences between the actual and expected concentrations of the sample solutions, mainly because the pesticide solutions did not reach 100% complete and homogeneous mixing, where some of the solutions have a lower drug content than expected, while some of them have a higher drug content than expected.

Fig 12 shows that the relative errors of the concentration of the sample solutions are negative in the range of mixing ratios from 1:1500–1:3000. This means that when mixing at low concentrations, the Bernoulli effect of the jet mixer causes a certain degree of upwards movement in the amount of solution supplied in the case of large-ratio mixing, despite the precise control of the solution by the pump in the test rig. When the mixing ratio is 300:1, the relative error of the sample solution concentration is less than 0.05. When the mixing ratio is 600:1, 900:1, 1200:1, the relative error of sample solution concentration is less than 0.08, but slightly higher than the error of the mixing ratio of 300:1. When the mixing ratio reaches 2000:1 and 3000:1, the relative error of sample solution concentration is larger, but each is less than 0.1. At this time, as the mixing ratio increases, the mass proportion of pesticides in the mixed solution decreases, and the content is less, which is more difficult to be completely mixed in water. Overall, when the mixing ratio changes, the relative error of the mixer is still relatively stable and basically within the range of ±0.1. Considering the relevant requirements, the results show that in the case of continuous operation, the mixer displays a stable temporal distribution and good uniformity of mixing.

#### Analysis of the spatial uniformity of mixing.

In order to ascertain whether the homogeneity of the well-mixed mixture undergoes any changes following its discharge from the mixer and during its transit through the pipeline until it is sprayed from the nozzle, a number of locations were selected from the plant protection spraying system for sampling. The homogeneity of the mixture was then analysed in terms of its spatial distribution.

In accordance with the test programme [2], to assess changes in the mixing ratio of pesticides and water, samples were collected at 10 different locations, and the sample concentrations at different mixing ratios were obtained after testing, as shown in Table 8. The unit is mg·L^-1^, and the changes in the relative error of the sample concentration are shown in Fig 13.

**Table 8:** Actual and expected concentrations of samples at different spatial locations for each mixing ratio.

Sampling point	300:1	600:1	900:1	1200:1	1500:1	2000:1	3000:1
**1**	9.537	4.658	3.139	2.428	2.159	1.599	1.101
**2**	9.548	4.666	3.151	2.438	2.131	1.587	1.091
**3**	9.547	4.668	3.153	2.439	2.133	1.585	1.089
**4**	9.548	4.666	3.151	2.438	2.131	1.587	1.091
**5**	9.558	4.673	3.218	2.443	2.115	1.568	1.085
**6**	9.561	4.679	3.221	2.451	2.124	1.552	1.081
**7**	9.556	4.678	3.173	2.466	2.114	1.564	1.077
**8**	9.554	4.675	3.174	2.465	2.113	1.565	1.076
**9**	9.561	4.679	3.213	2.455	2.101	1.574	1.099
**10**	9.558	4.683	3.215	2.453	2.103	1.573	1.095
**Expected concentration**	10	5	3.3	2.5	2	1.5	1
**CV (%)**	0.08	0.17	1.03	0.53	0.76	0.89	0.80

According to the data in Table 8 and Fig 13, the actual mass concentration and relative error exhibited different distributions at the sampling points at the different mixing concentrations. The data show that the differences in the locations of the sampling points lead to significant variations in the relative error at different mixing concentrations, and the overall relative error trend is similar to that of the temporal distribution, characterised by uniformity. Specifically, based on the mixing process from the mixer through the main pipeline to the distributor, spray bar and end spray nozzle, it was found that the relative error of the pesticide solution gradually decreased. And at the same mixing ratio, the relative errors of solutions sampled at different locations varied less than 0.03, proving good spatial distribution uniformity. By comparing the actual mass concentration and relative error at the first and last nozzles of the different spray bar segments, it was found that the values at the last nozzle were slightly smaller than the values at the first nozzle, suggesting that after the mixed solution was transported through the mixing system, further mixing of the solution occurred before spraying. The concentration uniformity of the mixed solution was better than the uniformity of the solution in the mixer. Moreover, according to the data in the table, at the selected 10 sampling points, the actual concentration of the two-stage injection mixer was generally higher than the expected value when mixing in large proportions, a trend similar to that regarding the uniformity of the temporal distribution of mixing, and the error decreased gradually with the extension of the pipeline and the spraying of the mixture from the terminal nozzle. Overall, the uniformity of the spatial distribution of the two-stage injection jet mixing system meets the relevant requirements for practical use.

The methods employed to assess the mixing efficacy of mixers in the field of plant protection are deficient in their consideration of the homogeneity of the mixture in the spatial dimension. The quantitative analysis methodology employed in this study takes into account the homogeneity of the distribution of the mixture in both the temporal and spatial dimensions, thereby yielding more compelling conclusions.

## Discussion

This study evaluated the homogeneity and mixing performance of a two-stage injection jet mixer during the mixing of pesticides with water. The study delves into the mixing effect at various ratios, analyzing the temporal and spatial distribution uniformity using high-speed photography and UV spectrophotometry. Data analysis of the test results by taking the average value of multiple repetitions of the test showed that the mixer had good mixing performance and good temporal and spatial distribution uniformity of the mixture in the range of mixing ratios from 300:1–3000:1.

Similar to the findings of Ou [13] and Mohammadreza [14] et al., this study also emphasizes the importance of optimizing nozzle dimensions and positions for efficient mixing. However, the innovative two-stage and injective design of the mixer integrates the advantages of both jet and injective mixers, enabling a wide mixing ratio range from 300:1–3000:1. By leveraging the injection mechanism, it achieves precise water supply and energy loss compensation, thereby enhancing the homogeneity of the mixture. The mixer demonstrates superior performance, high precision, rapid response, and eliminates the need for secondary pressurization during application. These advancements address the challenges of precision and stability in large-ratio mixing, representing a significant improvement in mixer performance optimization. Furthermore, this study validates the effectiveness of a dual-stage injective jet mixer under high mixing ratios, a domain underexplored in existing research.

In this study, the mixing performance of a two-stage injected jet mixer was comprehensively evaluated using both experimental and image analysis methods. Image analysis results indicated that the grey values of pure pesticide were more uniformly distributed in both axial and radial directions. Upon adding water, the grey scale value distribution of the mixture closely resembled that of the pure pesticide. The two-dimensional images of the mixture’s axial and radial profiles exhibited a distribution pattern similar to that observed for the pure pesticide, as reported by Dai [24]. This suggests that the two-stage injected jet mixer effectively promotes uniform mixing of pesticides and water. The root mean square error (RMSE) analysis revealed that as the mixing ratio increased, the percentage change in RMSE showed an upward trend with minimal variation [37]. This phenomenon can be attributed to the reduced pesticide concentration within the mixture, leading to decreased turbulence intensity during mixing, which affects mixing homogeneity. Nevertheless, the injected energy compensation mechanism keeps the RMSE within 10%, which is better than the 13.389% of the single-jet mixer designed by Song [7,38]. Specifically, when the water-to-pesticide mixing ratio was 300:1, the minimum error was 8.57%, while at a ratio of 2000:1, the maximum error was 9.94%. Overall, the error changes remained relatively stable, indicating that the RMSE percentages at different mixing ratios did not differ significantly, suggesting consistent and homogeneous mixing effects. Chen’s study examined the relationship between flow rates and RMSE percentages of the mixture, whereas this paper investigates the relationship between mixing ratios and RMSE percentages [7]. Despite the different parameters, the results are consistent, showing a similar trend. This consistency is likely due to the fact that as the flow rate increases, the volume of solution passing through a region per unit time also increases, but the pesticide supply rate remains constant, leading to a relative decrease in pesticide concentration at higher flow rates. Similarly, as the mixing ratio increases, the proportion of water in the mixture rises, resulting in a decrease in pesticide concentration. These findings indicate that both the water-to-pesticide mixing ratio and flow rate can influence the concentration of the mixture, aligning with Chen’s observations [7].

The temporal and spatial distribution uniformity of the mixer at different mixing ratios was analysed using UV spectrophotometry. Due to the short interval between sampling times in the experiment (30 seconds), this ensured that the samples taken reflected the mixing effect of the mixer over a short period of time. The findings revealed that the error range between the actual and expected mass concentration of the mixture sampled at different times was less than 0.1, indicating that the mixer exhibited superior temporal distribution uniformity and possessed the capacity to achieve uniform mixing of pesticides and water within a brief period, a feature of paramount importance for pragmatic plant protection operations. However, when the mixing ratio exceeds 1500:1, the mixture’s concentration increases, a consequence of the Bernoulli effect inherent in the jet mixer’s design when operating at lower concentrations. This effect leads to an upward trend in the supply of pesticides during large-ratio mixing, a finding that aligns with those reported by Xia [35]. At the same mixing ratio, the variation of error at different sampling points is less than 0.03, which indicates that the mixer has good spatial distribution uniformity. And with the extension of the pipeline, the more backward the sampling point is, the smaller the error of the mass concentration of the mixed solution is. This is because the further mixing of pesticide and water is promoted during subsequent solution delivery due to the zigzag changes in the pipeline and the secondary distribution of the valve body. Therefore, appropriately extending the length of the pipeline of the spraying system is conducive to improving the homogeneity of the mixture and reducing the tiny mixing errors caused by the mixing accuracy of the mixer as well as other irresistible factors to the extent that the pesticide is not homogeneous in the final sprayed solution.

The commonly used industrial jet mixers are usually designed with a single-stage structure, which has a narrow range of mixing ratios (generally 50:1–1000:1), and is prone to mixing inhomogeneity at high dilution ratios (>1000:1) and high pressure loss. In contrast, the two-stage jet mixer designed in this paper significantly widens the range of mixing ratios (300:1–3000:1) and maintains excellent mixing homogeneity (RMSE<10%) at ultra-low concentrations (0.03%) through an innovative two-stage tandem structure and injection compensation mechanism. At the same time, the design achieves a compact structure with no moving parts by optimizing the energy transfer efficiency, which provides advantages in mixing performance and stability, and meets the demand for wide-ratio, high-efficiency mixing in precision agriculture applications.

Therefore the two-stage injection jet mixer designed in this study can be applied to an upland gap planter for field plant protection spraying operations, and the real-time matching of the application volume and mixing ratio can be achieved through the precise control of the water injection system and the pesticide injection system. This study also has some limitations. At present, it mainly focuses on the mixing effect of water-soluble pesticides, and the mixing effect of other types of pesticides has not been studied in depth. In the future, we need to integrate various sensors and intelligent control algorithms to regulate the mixing ratio in real time and test the mixing compatibility of pesticides such as powder and emulsion.

## Conclusion

In this paper, a two-stage injection jet mixer was designed and tested for mixing pesticides with water in a wide ratio (300:1–3000:1), and its mixing homogeneity was verified by image analysis method and UV spectrophotometry. The main conclusions are as follows:

1. CFD optimization showed that the structural parameter design of the mixer significantly enhanced the turbulent dissipation efficiency, which not only ensured the homogeneous mixing of the pesticide and the water, but also ensured the stability of the mixing process.
2. The root mean square error (RMSE) of the mixture was stable at 8.57%−9.94% over the range of mixing ratios from 300:1–3000:1, indicating that the mixer could maintain high homogeneity under extreme dilution conditions.
3. UV spectrophotometry showed that the relative error between the actual mass concentration and the expected value was less than 0.1%, confirming the temporal and spatial distribution homogeneity of the mixture and the reliability of the mixer in continuous operation.

The mixer designed in this study has a wide range of mixing ratios and can be adapted to diverse agricultural scenarios to meet the demand for precision variable spraying. In the future, the compatibility of multiphase mixtures (e.g., emulsions, suspensions) will be further investigated, and smart sensors will be integrated to realize real-time mixing ratio regulation.

This study provides a reliable technical solution for accurate online mixing of pesticides, which is of great significance for promoting sustainable agricultural practices.
